# Prosthetic Knee Joint Infection Due to *Candida lusitaniae:* A Diagnostic and Therapeutic Odyssey: A Case Study

**DOI:** 10.3390/diagnostics12112640

**Published:** 2022-10-31

**Authors:** Vasileios Giovanoulis, Angelo V. Vasiliadis, Christos Koutserimpas, George Samonis, Cécile Batailler, Tristan Ferry, Sébastien Lustig

**Affiliations:** 1 Orthopaedic Surgery and Sports Medicine Department, Croix-Rousse Hospital, Lyon University Hospital, 69007 Lyon, France; 2 2nd Orthopedic Department, General Hospital of Thessaloniki “Papageorgiou”, 56403 Thessaloniki, Greece; 3 Department of Orthopaedics and Traumatology, “251” Hellenic Air Force General Hospital of Athens, 11525 Athina, Greece; 4 Department of Internal Medicine, University of Crete, 71500 Heraklion, Greece; 5 Department of Medicine, University of Lyon, 69622 Lyon, France; 6 Department of Infectious Diseases, Groupement Hospitalier Nord, Hospices Civils de Lyon, 69004 Lyon, France; 7 Regional Reference Center for the Management of Complex Bone and Joint Infections, CRIOAc Lyon, Hospices Civils de Lyon, 69004 Lyon, France; 8 Centre International de Recherche en Infectiologie (CIRI), Inserm U1111, Université Claude Bernard Lyon 1, CNRS UMR5308, Ecole Normale Supérieure de Lyon, 69007 Lyon, France

**Keywords:** *Candida*, prosthetic joint infection, total knee replacement, deep fungal infection, arthroplasty infection, arthroplasty complications

## Abstract

Prosthetic joint infections (PJIs) caused by fungi, although relatively rare, represent a major surgery-related complication. An extremely rare fungal PJI, following revised total knee replacement (TKR) caused by *Candida lusitaniae*, is reported, and a meticulous review of similar cases is provided. A 74-year-old female, who underwent primary total knee arthroplasty 10 years ago and a revision surgery three weeks ago, presented with signs and symptoms of PJI. *C. lusitaniae* was eventually isolated from the periprosthetic tissue using the MALDI-TOF VitekMS–bioMérieux technique. Multiple strategies for managing this fungal PJI were performed, and finally, the patient was treated successfully with an intramedullary arthrodesis system and proper antifungal treatment, including fluconazole. A multidisciplinary approach is essential for the diagnosis and treatment of such severe infections. In persistent cases and in cases where revision surgery is extremely difficult to perform, arthrodesis seems to be an effective solution for the elimination of the infection. The efficacy of the therapeutic management of fungal PJIs remains unclear. Therefore, more research should be reported, focusing on proper treatment so that the optimal strategy in treating these severe infections may be established.

## 1. Introduction

The number of patients undergoing a total joint replacement of the hip and knee has increased rapidly worldwide over the last few decades, and it is projected to rise considerably over the next five to 10 years [1]. Throughout the years, joint arthroplasty has rapidly witnessed major evolution. In the past, surgeons used acrylic cement and low-friction prostheses, while surgeons now utilize robotic and/or navigation platforms, and fast-track and transfusion reduction protocols have become common practice [2,3,4,5]. Nevertheless, complications have not been completely eliminated, with periprosthetic joint infections (PJIs) being among the most challenging complications of joint replacement surgeries and accounting for 1% to 3.7% of all cases [6,7,8,9]. This incidence is even higher for patients who have undergone reconstruction surgery in multiple joints [10,11]. A plethora of pathogens may cause PJI, including, most commonly, gram-positive and gram-negative organisms and anaerobic bacteria, and more rarely, fungi [6,8,9].

There have been several risk-factors associated with PJIs, such as obesity, smoking, diabetes mellitus, the experience of the surgeon, the patient’s gender (males are more prone to PJIs), the patient’s general health status, the substitution of the patella, the surgery’s duration, and a prolonged hospitalization [12].

Fungal PJI represents a rare infection, occurring in approximately 1% of the total number of infected joint replacement cases, with *Candida albicans* being the most commonly responsible fungus, followed by non-*albicans Candida*, including *C. glabrata*, *C. parapsilosis*, *C. freyschusii*, and *C. lusitaniae* [7,11]. It should be noted that there has been a reported rise in the number of deep fungal infections over the last decade, which can be mainly attributed to the elderly population, as well as the rise of immunosuppressed hosts [13,14].

Immunosuppression, malignancy, tuberculosis, other severe underlying systemic illnesses, a prolonged hospitalization, and the previous use of antimicrobials represent risk factors associated with infections caused by *Candida* species [15].

Scarce data exist regarding the management of fungal PJIs and management outcomes due to the rarity of these infections. In everyday clinical practice, a two-stage revision arthroplasty (TSRA) combined with a prolonged antifungal treatment (AFT) represents the prevailing therapeutic approach [16]. PJIs in already revised knee arthroplasties represent an additional challenge, since re-revision surgery is an extremely demanding and complicated procedure that poses unique surgical and medical challenges. Other options for PJI eradication, especially in cases where revision reconstruction surgery is not possible, are arthrodesis and amputation.

An extremely rare PJI case following revised total knee replacement (TKR) caused by *C. lusitaniae* is presented. Thorough details regarding the diagnosis and the therapeutic management are analyzed. A meticulous review of similar cases is also provided, discussing the applied diagnostic and treatments methods. The aim of the study is to present the current diagnostic and therapeutic options of such severe infections and to highlight the challenges of diagnosing and treating these patients.

## 2. Case Presentation

In February 2022, a 74-year-old female with a Body Mass Index of 23.1 kg/m² was evaluated in a university outpatient orthopedic clinic for hip and knee reconstructive surgery for her right knee prosthetic joint complex situation.

**Figure 1:**
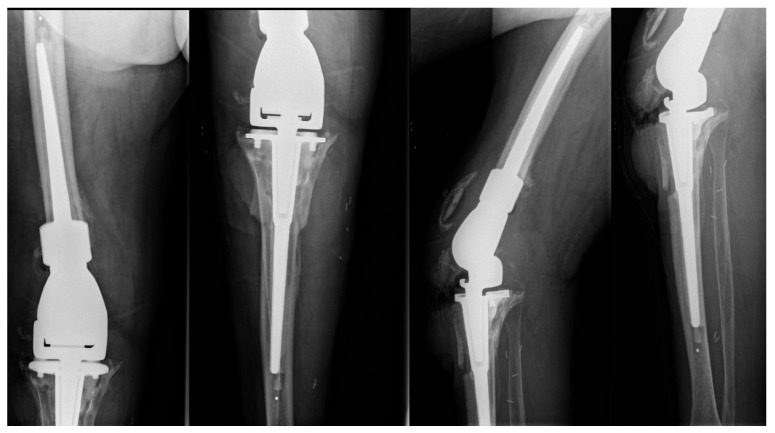
Anteroposterior and lateral X-ray views of the revised arthroplasty, which was performed prior to the patient’s presentation in the outpatient clinic.

The patient had undergone right knee arthroplasty due to osteoarthritis in 2012. The patient also suffered from type 2 diabetes mellitus and hypertension, and while she had also undergone TKR of the left knee in 2018, she presented without any problems during the current visit. A revision in the right knee with a resection type arthroplasty (Stanmore Mets^®^) had been performed in November 2021 due to a mechanical failure of the prosthesis, which was related to oversized implants associated with anterior tibial tubercule osteotomy (Figure 1).

Three weeks after the revision surgery, skin necrosis was observed, which led to a debridement of the wound and later to a restoration with a medial gastrocnemius flap. Intra operative tissue samples were cultured, and they yielded methicillin-resistant *Staphylococcus aureus* (MRSA), methicillin-resistant *Staphylococcus epidermidis* (MRSE), and *Morganella morgani.* The patient had been treated with linezolid and ceftriaxone. Two weeks after the initial flap placement, a new debridement was performed due to the unsuccessful wound healing associated with the flap removal.

Upon presentation, she was afebrile, in good general condition, and hemodynamically stable. Her skin presented with proximal necrosis and sinus tract, while a loss of the extensor knee mechanism was present. The skin flap also had proximal wound disunity, and the prosthesis was exposed. Laboratory examination revealed C-reactive protein (CRP) = 27 mg/L and white blood cells (WBC) = 6.4 G/L.

Following thorough discussion with the specialized and multidisciplinary team at the complex bone and joint infections center called CRIOAc Lyon (http://www.crioac-lyon.fr, accessed on 20 August 2022), a ‘two-stage revision’ with the removal of the prosthesis, a patellectomy, and the implant of a long cement stem spacer impregnated with gentamycin and vancomycin and a sartorius muscle flap was performed. Intraoperative samples yielded *Enterococcus faecalis* and *Corynebacterium pseudodiphtheriticum*, and she was commenced on daptomycin 350 mg/day and piperacillin/tazobactam 4 g/8 h/day.

**Figure 2:**
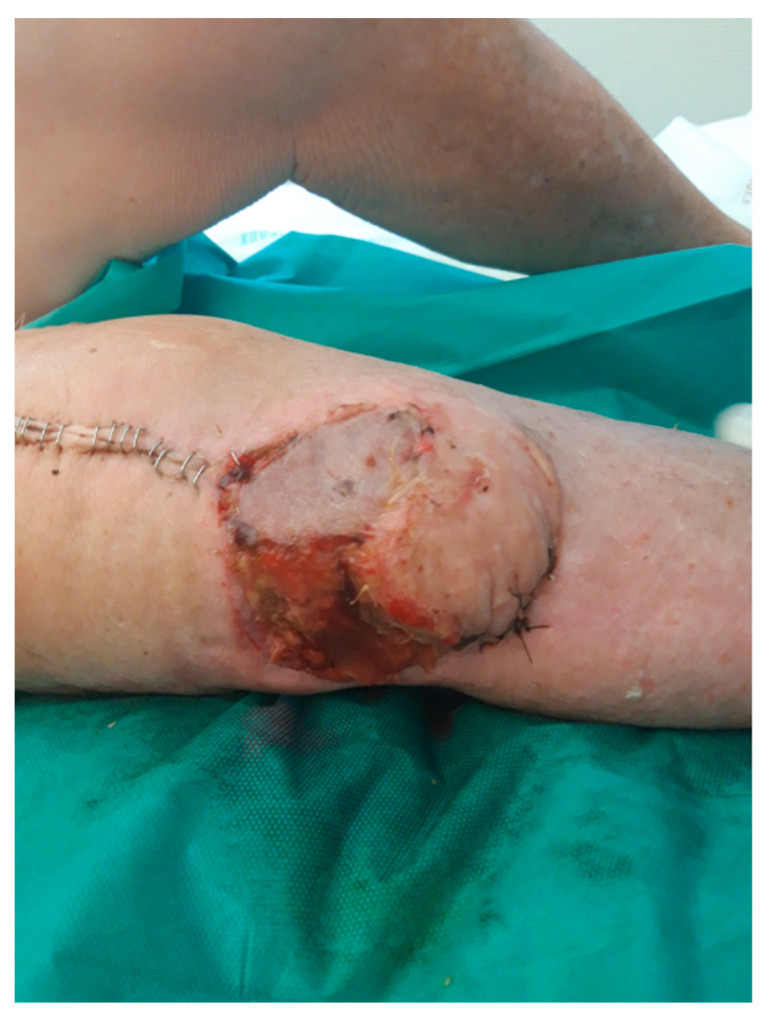
The patient developed a wound healing disorder (skin necrosis) anterolateral to the operational area (knee joint).

**Figure 3:**
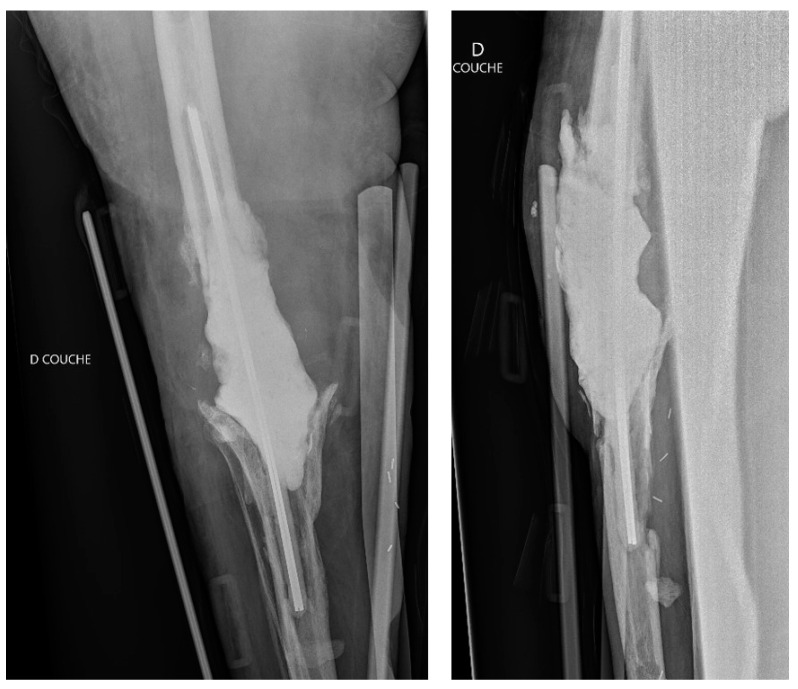
Anteroposterior and lateral post-operative radiographic views of the right knee after component explanation with an antibiotic-loaded cemented spacer, fixed with two metalic rods.

Three weeks later, the evolution of the flap was not favorable, with 25% skin necrosis (Figure 2). At that point in time, CRP was 29 mg/L and WBC were 6 G/L. A lavage, surgical debridement and a change of the spacer impregnated with antibiotics (gentamycin and vancomycin) was performed at that point (Figure 3), while a new lateral gastrocnemii flap was put in place.

Peri-prosthetic cultures yielded *Candida lusitaniae,* as well as *Staphylococcus capitis* and *Klebsiella oxytoca*. The technique used was MALDI-TOF VitekMS–bioMérieux. The patient was commenced on intravenous daptomycin 350 mg, piperacillin-tazocillin 4 g/8 h, and caspofungin 70 mg one/day. The isolated *Candida* was susceptible to caspofungin (MIC 0.04 mg/L), fluconazole (MIC 0.064 mg/L), and micafungin (MIC 0.047 mg/L).

She was hospitalized for another two weeks, receiving intravenous antimicrobial and AFT. During hospitalization, she remained afebrile and hemodynamically stable. Upon discharge, the patient had no symptomatology related to PJI, while laboratory findings showed WBC = 6.16 G/L and CRP = 6.8 mg/L.

**Figure 4:**
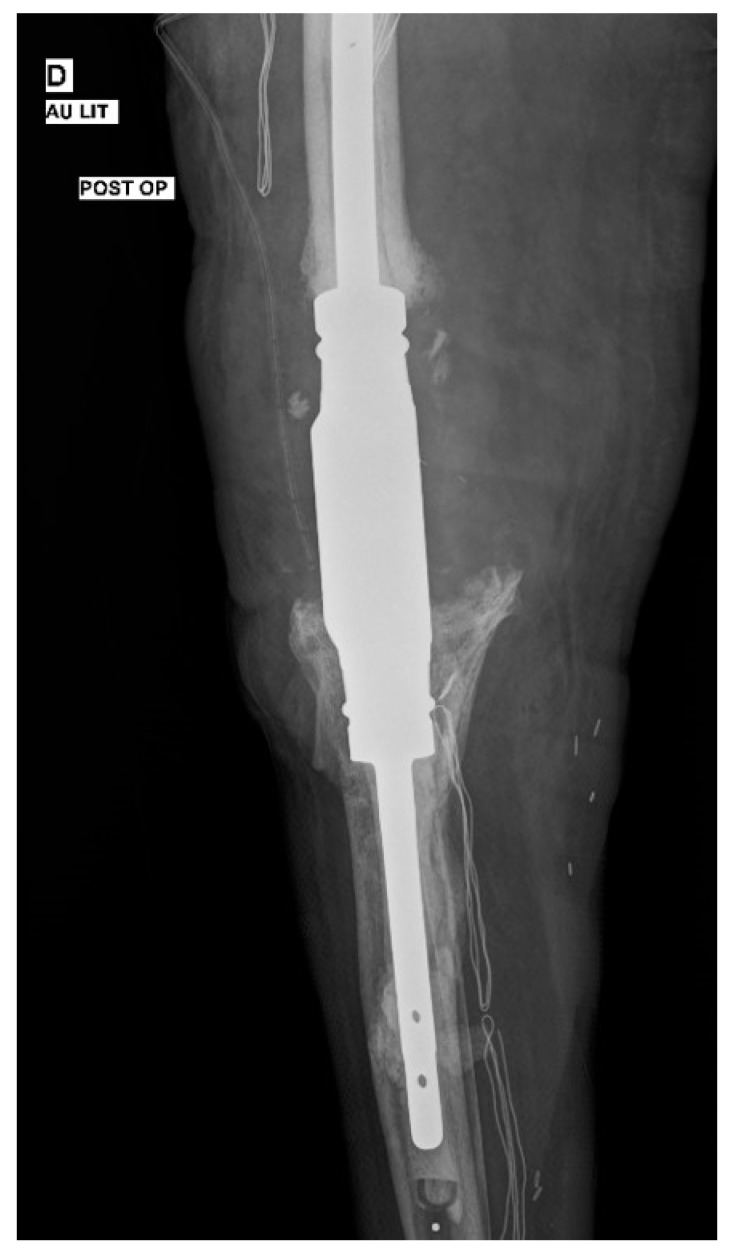
Anteroposterior post-operative radiographic view of a prosthesis-arthrodesis implant.

She received per os ceftaroline 600 mg/12 h, daptomycin 350 mg/24 h, and fluconazole 400 mg/24 h following a loading dose of 800 mg. The patient was followed up with for eight more weeks. No relapse was observed after the final surgical procedure. At this point, arthrodesis of the knee joint was decided. A prosthesis-arthrodesis (Mutars^®^ RS implantcast) was inserted (Figure 4), while the new intra-operative samples yielded no microorganisms or fungus.

**Figure 5:**
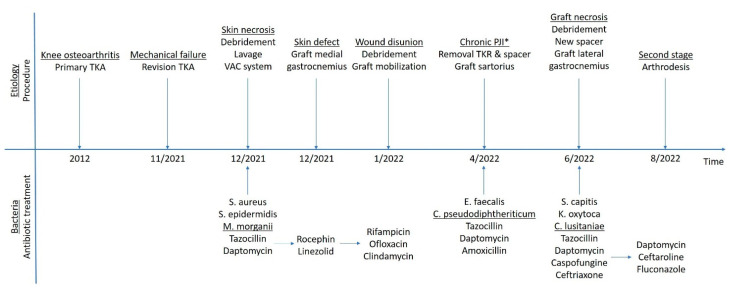
An overview of the patient’s clinical course. (*) represents the point in time that the decision to remove the implants was made.

Four months after the last operation, she was free from pain and had no signs of PJI. At that point, antimicrobials and AFT were discontinued. An overview of the patient’s clinical course is summarized in Figure 5. At the seven-month follow up, she was in good general health, independent in her everyday life, and she had no signs or symptoms of an infection.

## 3. Discussion

Despite the fact that complications associated with TKR are rare, they vary from minor issues to catastrophic ones and life-threatening situations as well. The incidence of some of these complications may be decreased if TKR is performed in properly equipped hospitals by experienced orthopaedic surgeons [17,18]. Attempts should be made to diminish the risk of such complications with suitable patient selection, thorough surgical technique, and meticulous postoperative treatment. PJI represents a devastating complication related to TKR, and it is responsible for most revision arthroplasties [17].

Fungal PJIs represent rare clinical entities, and non-*albicans Candida* fungal PJIs are very rarely reported [7,8]. Diagnostic and therapeutic methods require a multidisciplinary approach [11]. The management of these infections is demanding and challenging [7,8,11].

PJI represents one of the most common reasons for revision surgery [16]. PJIs are defined as early onset (<3 months following initial operation), delayed onset (3–12 months following operation), and late onset (>12 months following operation) [9]. According to this definition, the PJI in the reported case could be described as early onset. The incidence of PJI in TKR is estimated to be approximately 1–2%, and an abundance of measures have been proposed to eliminate such severe orthopaedic infections [19,20].

TKR has a greater risk of PJI compared to hip arthroplasty. This has been attributed to the knee joint having a higher range of motion and the proximity of the joint to the skin, with lesser soft tissue coverage [9,15]. The number of reconstruction joint surgeries rises each year, along with the number of immunocompromised hosts and the increasing use of invasive devices, such as central venous catheters; thus, fungal PJIs are expected to increase [7,12,21]. Data regarding AFT, AFT duration, the type of surgical interventions, and infection outcomes are scarce and have to be further clarified for better medical care of such patients.

This study has exhibited, in detail, the diagnostic and therapeutic challenges that such a severe infection possesses, especially in cases of revised knee arthroplasty. Non-*albicans Candida* PJIs represent a rare clinical entity; hence, they should be reported for the evaluation of diagnostic and therapeutic options. The reported patient suffered PJI in a revised TKR due to *C. lusitiniae,* and she was finally treated successfully with knee arthrodesis and prolonged proper AFT. PJIs due to fungi in already revised joint surgeries have increased morbidity and possess special characteristics regarding, mainly, surgical management.

Hematogenous spread represents the most common mechanism of *Candida* deep tissueinfection (67%), followed by direct inoculation (25%), and contiguous infection (9%) [8,11,21]. The most frequently reported species is *Candida albicans*. Nevertheless, deep fungal infections’ incidence due to other non-*albicans Candida* species is increasing [7,11]. A possible explanation for the reported PJI case could be direct inoculation, since signs of the infection were evident just three weeks after the revision knee reconstruction surgery. Fungal intraoperative contamination should be considered in these cases. However, no similar infections have been reported from the same institution during a prolonged time-period. Furthermore, the operating theaters and the sterilization department are regularly disinfected. It should also be noted that each revision joint surgery raises the incidence of PJI, while iterative antimicrobial prescriptions may lead to fungal colonization of the skin, which may then lead to deep fungal post-operative infections [11,15].

Following a thorough electronic literature search of the PubMed and MEDLINE databases, only four other cases of PJIs due to *C. lusitaniae* in revision knee arthroplasties were identified [22,23,24,25]. Table 1 highlights the main features of these cases. Three of these cases involved the knee joint (75%), while bacterial co-infection existed in two of those (50%). In the reported case, bacterial co-infection was also present. It should be noted that coexisting bacterial infection has been documented in about 15–20% of fungal PJIs [11,12,26]. Bacteria and fungi have synergistical action within the prosthetic biofilm, which leads to more severe infections [26]. PJI caused by more than two co-infective organisms, such as the reported one and, especially, multidrug-resistant ones, have been documented to possess a higher risk of recurrent infections [11,26].

Immunosuppression, systematic diseases, and long-term antimicrobial use are among the several risk factors identified in deep fungal infections [15]. Other documented invasive *Candida* species infection risk factors are diabetes mellitus, catheters, abdominal surgery, coexisting or previous bacterial PJI, and silent Candida bacteremia [7,11,15]. This patient suffered non-insulin-dependent diabetes, which could be considered as a risk factor for invasive *C. lusitanae* infection. Diabetes mellitus has already been documented as a major predisposing factor for deep fungal infections. Unfavorable mycotic infection outcomes have been associated with uncontrolled diabetes [26,27]. Additionally, diabetes mellitus leads to immunosuppression by impaired innate and acquired immunity. Functions of neutrophils, such as phagocytes, chemotaxis, and cytokine-production are decreased in diabetes, while hyperglycemia and Th2-axis shift reducing Th1-dependent immunity are observed in patients suffering from diabetes mellitus [27,28].

In addition, the reported patient had undergone multiple surgical interventions, and she had received repeated cycles of antimicrobial treatment, which could have predisposed her to the *C. lusitanae* infection, since the prolonged use of antimicrobial agents represents an iatrogenic risk factor for invasive candidiasis due to the alteration of the patient’s normal flora [15,17].

It should be noted that prophylactic AFT has not yet been recommended in any high-risk cases [16]. However, considering the increased incidence of immunocompromised patients undergoing joint reconstruction surgery, it is evident that this should be investigated. Likewise, cement impregnated with antifungal agents could also be used therapeutically and/or prophylactically in some high-risk patients. The literature lacks information and data regarding antifungal-impregnated cement for the therapeutic management of PJIs caused by fungi, and no commercially available cement producing AFT exists [29]. A few cases report that different antifungal agents may be topically released in vivo. Nevertheless, the proper impregnation type and dosage of antifungal agents have not yet been defined [29].

*C. lusitiniae* is an environmentally ubiquitous ascomycetes yeast with an unknown specific ecological niche [30]. It is of the utmost importance to report the epidemiology and, especially, the management of these cases in order to better comprehend the therapeutic options and outcomes of these severe infections.

Regarding the diagnosis of PJI, initial imaging includes plain X-rays that could possibly reveal signs of prosthesis loosening and sequestrum. Inflammatory markers, including CRP and erythrocyte sedimentation rate, are also helpful for the diagnosis and during the course of the disease [31]. Thereafter, diagnostic arthrocentesis may be performed [16,30]. Regarding intraoperative tissue-specimens, at least three samples (ideally five) should be sent for microbiological and histological examination. [31]. In the present case, the causative fungus was cultured from intraoperative periprosthetic tissue specimens, and the technique used was MALDI-TOF VitekMS–bioMérieux. Mass-spectrometry assay represents a modern method of fungus’ identification [32,33,34]. Particularly, the performance of MALDI-TOF technology may accurately identify fungus and, more specifically, *Candida* species [33]. Thus, the use of MALDI-TOF MS-based fungus analysis may shorten the time needed to confirm the diagnosis, allowing clinicians to begin the proper AFT in a timely manner.

Optimal treatment for Candida PJIs has been controversial. It seems that two TSRA represents the gold standard surgical procedure, combined with prolonged proper AFT [16]. Several other surgical options to control such fungal infections have been proposed, such as debridement and retention of the prosthesis, one stage revision arthroplasty (OSRA), systemic antifungal lifetime suppression therapy with prosthesis retention, resection arthroplasty with reimplantation or amputation, and arthrodesis [7,12,21]. It is noted that the most used surgical procedures, such as arthrodesis and amputation, although proven to be life-saving therapeutic approaches, crucially affect the patient’s life quality [35,36,37]. The reported case underwent arthrodesis due to the massive bone defects, the extensor mechanism insufficiency, and the need to eradicate the severe infection.

Regarding surgical treatment of PJI due to *C. lusitanae*, Klatte et al. reported one case treated with OSRA, which could be considered a safe procedure, while Hwang et al. and Viotti et al. have successfully managed two cases with TSRA [23,24,25].

Despite advances in antimicrobial and antifungal agents, some knee PJIs remain resistant to the proper treatment of causative organisms [37]. There are case series reporting an infection recurrence rate of up to 28% after revision TKR due to PJI [37,38]. In these infection cases that are extremely difficult to eradicate, there are two surgical options: above-the-knee amputation and arthrodesis [35,36,37,38]. The reported case represents a characteristic example of such a case due to the significant bone defects after the previous revision and the severe skin lesions, requiring skin flaps. While these operations have a huge negative effect on the patient’s life quality, they also usually manage to control the infection.

Table 2 highlights AFT of all published PJI cases due to *C. lusitiniae.* Regarding the preferred AFT, two cases were treated with a single agent (Voriconazole or Micafungin), and one case was treated with two antifungal agents (Amphotericin B + fluconazole). The treatment duration varies generally according to the clinical and laboratory findings. The reported case received caspofungin for two weeks and then fluconazole for four months.

It is of note that the type and duration of AFT remains controversial. In cases of fungal native joint septic arthritis, the Infectious Diseases Society of America recommends 400 mg fluconazole every day for a total of six weeks or an echinocandin, such as caspofungin, for a total of two weeks, followed by 400 mg fluconazole daily for at least four weeks. It should be noted that the lipid formulation of amphotericin B for two weeks, followed by fluconazole for at least four weeks, represents a less than preferable alternative [16,39]. However, in PJI cases, implants are involved; hence, these guidelines do not apply per se. Very limited data exist regarding fungal PJIs; therefore, no clear therapeutic consensus exists. Regarding the adverse effects of these agents, fluconazole has been linked to hepatotoxicity, while amphotericin B is relatively nephrotoxic, limiting its prolonged use [40,41,42]. Fluconazole does not have serious adverse effects. Furthermore, this agent has shown favorable pharmacokinetic characteristics of rapid oral absorption with high bioavailability; an extended half-life, allowing once per day administration; and a high concentration of this antifungal in joint fluid, similar to that in plasma [16].

## 4. Conclusions

PJIs represent a major cause of morbidity following total joint replacement. The most common risk factors for developing PJI include advanced age, obesity, and the presence of comorbidities causing immunosuppression, such as diabetes mellitus, and prolonged antimicrobial treatment alters the host’s flora, which also attributes to deep fungal infections. A multidisciplinary approach is of the utmost importance for the diagnosis and the management of this severe infection. The combination of TSRA and prolonged proper AFT seems to be the therapeutic option of choice, while in persistent cases, including those with significant bone defects where revision surgery is extremely difficult to perform, arthrodesis may prove beneficial in eradicating the infection. Nevertheless, studies with long follow-up are needed to establish such a conclusion, since there is a possibility of chronic infection around the arthrodesis implant. Often, the outcomes of management remain unclear; hence, these cases should be reported, while additional data and further research are essential, mainly regarding proper therapeutic management and/or prophylaxis strategies so ideal policy about treating these severe infections may be established.

## Figures and Tables

**Table 1:** Summary of included studies.

Study	Gender/ Age	Joint	Bacterial Co-Infection	CRP (mg/L)	ESR (mm/h)	Comorbidities	Number of Revisions	Time from Implantation to Symptoms’ Onset (Months)	Time from Symptoms Onset to Diagnosis (Months)
Present study, 2022	F/74	Knee	*K. oxytoca* *S. capitis* *C. lusitaniae*	29	-	DM, HTN	3	1	1
Saconi et al., 2020 [21]	M/63	Hip	No	-	-	Kidney transplantation	-	-	-
Viotti et al., 2019 [22]	F/79	Knee	-	8.4	118	RA	-	120	-
Klatte et al., 2014 [23]	M/74	Knee	*S. aureus* *S. mitis*	>22	-	-	2	17	6
Hwang et al., 2012 [24]	F/66	Knee	NR	4.3	29	-	-	-	48

Abbreviations: M, male; F, female; K. oxytoca, Klebsiella oxytoca; S. capitis, Staphylococcus capitis; C. lusitaniae, Candida lusitaniae; S. aureus, Staphylococcus aureus; Streptococcus mitis, S. mitis; CRP, c-reactive protein; ESR, erythrocyte sedimentation rate; RA, rheumatoid arthritis; DM, diabetes mellitus; HTN, hypertension; and (-), not reported.

**Table 2:** Management of reported PJI cases due to *Candida lusitaniae*.

Study	ST	Time between Stages in TSRA (Months)	AFT	AFT (Duration)	Follow-Up (Months)	Outcome
Present study, 2022	Arthrodesis	-	Fluconazole	4	4	Success
Saconi et al., 2020 [1]	OSRA	-	Micafungin, fluconazole	24	Lost	-
Viotti et al., 2019 [13]	TSRA	11	Micafungin	6	18	Success
Klatte et al., 2014 [19]	NS	-	Voriconazole	>2	30	Success
Hwang et al., 2012 [20]	TSRA	3	Amphotericin B, then oral fluconazole	-	43	Success

Abbreviations: ARC, antimicrobial regimen in cement; AFT, antifungal treatment; ST, surgical treatment.

## Data Availability

Not applicable.
